# Laryngeal photobiomodulation: application sites, interferences from body mass index and skin phototype

**DOI:** 10.1590/2317-1782/20242023333en

**Published:** 2024-08-02

**Authors:** Elisa Meiti Ribeiro Lin Plec, Viviane Souza Bicalho Bacelete, Marco Aurélio Rocha Santos, Ana Cristina Côrtes Gama

**Affiliations:** 1 Programa de Pós-graduação em Ciências Fonoaudiológicas, Departamento de Fonoaudiologia, Faculdade de Medicina, Universidade Federal de Minas Gerais – UFMG - Belo Horizonte (MG), Brasil.; 2 Departamento de Fonoaudiologia, Faculdade de Medicina, Universidade Federal de Minas Gerais – UFMG - Belo Horizonte (MG), Brasil.

**Keywords:** Anatomy, Larynx, Low Level Light Therapy, Voice, Laser Therapy

## Abstract

**Purpose:**

Establish points on the neck, correspondent to the laryngeal topography, where to apply Low Level Light therapy (LLLT), to evaluate the incidence of light through variables such as skin phototype and body mass index (BMI).

**Methods:**

This is a cross-sectional, analytical, observational study, carried out with 15 vocally healthy women, between 18 and 50 years of age, who were divided into three groups, according to BMI and skin phototype. Six anatomical reference points were established to locate the larynx and its musculature, with visual monitoring by videonasolaryngoscopy, to assess light reach (present/absent) and degree of illumination (from very weak to very strong) in the larynx during the LASER application at doses of 3J, 6J and 9J. A flexible endoscope was used for visual monitoring during the LASER application, and subsequent image analysis.

**Results:**

The light reached the larynx at doses of 3J, 6J and 9J, in the anterior commissure of the vocal folds, membranous (thyroarytenoid muscle) and cartilaginous portions of the vocal fold and the cricothyroid muscle. The degree of LASER light illumination decreased in overweight and obese participants and increased in moderate brown and dark brown skin phototypes.

**Conclusion:**

Data suggest that the LLLT penetrates differently according to skin phototype and BMI, being more evident in individuals with Fitzpatrick IV and V phototypes and less evident with higher BMI levels. The evidence that the LASER light reaches the larynx in specific anatomical points provides direction for the standardization of its use in voice practice.

## INTRODUCTION

Photobiomodulation is defined as a form of light therapy that uses Light Amplification by the Stimulated Emission of Radiation (LASER), Light Emitting Diode (LED) and broadband sources that emit wavelengths in the visible and infrared spectrum^(1,2)^. This is a non-thermal process that causes photophysical and photochemical events at various biological scales. It is believed that light absorption occurs mainly in the mitochondria during the process of cellular respiration, accelerating intracellular ATP (adenosine triphosphate) synthesis^(1,3)^. In this therapy, light increases mitochondrial membrane potential and energy synthesis with a response peak between three and six hours after stimulation^(4)^.

Wavelength is the most important consideration in photobiomodulation, since without absorption there can be no interaction with the target tissue. For the incident radiation, the reflection is 4-7%, and in the remaining 93-96% that penetrate the skin, there is scattering or absorption, and as the used light wavelength increases, there is a corresponding increase in penetration depth. The absorption and scattering coefficients of living tissue are higher at lower wavelengths, though infrared light penetrates deeper^(5-7)^.

Using light as a means of treatment for several pathophysiological conditions has gained the attention of researchers in recent decades, with advances in the treatment of a variety of clinical conditions, causing analgesia, healing of skin and oral wounds, improvements to muscle performance, strength gain, fatigue, and inflammation reduction^(8,9)^.

The applicability of Low Level Light therapy (LLLT) as a rehabilitation method is beginning to be studied by speech therapists and otorhinolaryngologists. In a study in the field of voice, LED irradiation effects were investigated in the treatment of vocal fatigue in 16 vocally healthy individuals. Kagan et al.^(10)^ analyzed all the participants after a vocal loading test that consisted of reading out loud from a book as if they were in a large room, without amplification, for 15 minutes, followed by 5 minutes of rest. The reading task was repeated three times. The examiner monitored the subjects to maintain a target intensity range of 75-90 dB during phonatory loading. The analyzed data showed improvement in the acoustic and aerodynamic measurements and in the self-reported vocal effort when using the red wavelength one hour after the procedure. Some studies using in vitro and in vivo rabbit larynxes^(11)^, in vitro human larynxes^(12)^, and rat larynxes^(13)^, showed an increase in cell proliferation and migration in the vocal folds (VF), in addition to genes involved in the healing process, suggesting that photobimodulation is capable of modulating inflammatory and healing processes in laryngeal tissues^(8,10-13)^.

Considering that the larynx is an anatomical complex, it is necessary for the light energy to reach the target tissues to interact with laryngeal structures. Therefore, it is imperative for the luminous radiation to penetrate all anatomical layers from the skin, subcutaneous tissue, cervical muscles, cervical fascias, thyroid cartilage until reaching the intrinsic muscles of the larynx and vocal folds mucosa. Although photobiomodulation is clinically used in the treatment of dysphonia and to improve vocal performance, there are no studies that clearly define LLLT application sites in the larynx or which demonstrate that light is capable of penetrating all the neck structures up to the surface of the vocal folds.

During LASER penetration from the surface layers of the skin to the deep layers of the dermis and muscles, the light encounters mechanisms that serve as defense and barrier to prevent damage to the body. Two mechanisms that could influence the action of light on tissues are their thickness and physical barriers, such as melanin present in the epidermis^(14,15)^.

Today, the Body Mass Index (BMI), which is the result obtained by calculating: height/(weight)^2^, is widely used to predict obesity and its related cardiovascular risks. BMI is also proportional to the fat deposit, correlating with subcutaneous tissue thickness. The two main cutoff points in this calculation are those that define overweight when BMI 25-29.9 kg/m^2^ and obesity ≥ 30 kg/m^2 (15,16)^.

Another physical barrier produced by humans is melanin, present in surface epithelial cells which absorb light. Melanin has the following functions: photoreception, thermoregulation and photoprotection^(14,17)^. As to skin phototype and its sensitivity to sunlight, Fitzpatrick^(18)^ classified basic skin colors into six phototypes (SPT): Phototype I, basic skin color: white, in sunlight exposure burns easily, does not tan; Phototype II, basic skin color: white, burns easily, tans with difficulty; phototype III, basic skin color: light brown, may initially burn when exposed to the sun, but tans easily; phototype IV, basic skin color: moderate brown, hardly burns when exposed to sunlight, tans easily; phototype V, basic dark brown skin color, usually does not burn, tans easily; phototype VI, basic skin color: black, does not burn, darkens when exposed to sunlight. It is known that sunburn depends on the amount of energy from ultraviolet rays and the individual’s susceptibility. The Fitzpatrick scale is a simple way of inferring skin resistance to the effects of light. Thus, low-level LASER could have its effects changed by the different skin phototypes, associated with the physical barrier resulting from the existing melanin in the epidermal cells, a factor that may influence the need to adjust the LASER dosage for each individual^(17,18)^.

In order to advance in the construction of our scientific evidence on the effectiveness of LASER in the treatment of dysphonia, it is essential to establish anatomical points on the neck for the application of LLLT, as well as evaluate the reach of light in the larynx.

Therefore, our goal with this study was to establish anatomical points on the neck for the application of LASER in the larynx and evaluate the incidence of light through variables such as skin phototype and body mass index. The anatomical points were defined based on evaluating the range of red LASER light (present/absent) and the degree of illumination (very strong, strong, weak and very weak), at doses of 3J (Joules), 6J and 9J, aiming to standardize points in the neck and their relationship with the intrinsic muscles of the larynx, through visual monitoring by flexible nasofibrolaryngoscopy, the interference caused by skin phototype and body mass index. Due to the small number of participants, this study aims to give preliminary data to support the use of the LASER in the vocal field.

## METHODS

This is a cross-sectional, analytical, observational study carried out with 15 Brazilian women between 18 and 50 years of age, with neutral vocal quality, without vocal/laryngeal symptoms or complaints, defined based on speech-language and otorhinolaryngological evaluations, with different skin phototypes, classified by the Fitzpatrick scale^(17,18)^ and body mass index (BMI) classified as ranging between 18,5 and 25, or equal to/greater than 25^(15,16)^.

This study was approved by the Research Ethics Committee of the Federal University of Minas Gerais (UFMG), under the number 4.704.038 and all participants signed the Free and Informed Consent Form.

The participants were divided into three groups, with five participants in each group, according to skin phototype and body mass index (BMI). All the participants underwent otorhinolaryngological and speech-language evaluation. Women without laryngeal and vocal alterations were included.

For group I (G1), the inclusion criteria were considered for participants with skin phototypes I to III (light to light brown skin) and BMI ranging between 18,5 and 25 (min = 19,7; max = 25; mean = 22,4; SD = 1,86). Therefore, G1 was made up of five light to light brown skin and normal weight women, aged 19 to 31 years (mean = 24.4; SD = 5) .

For group II (G2), the inclusion criteria adopted were skin phototype from I to III (light to light brown skin) and BMI above 25 (min = 29,5; max 36,7; mean = 32; SD = 9,2). G2 was formed by five women aged 25 to 50 years (mean = 37.2; SD = 10.8), and the controlled independent variable was related to BMI (overweight).

In group III (G3), the inclusion criteria were skin phototype IV to VI (moderate brown to black skin) and BMI ranging between 18,5 and 25 (min = 20,5; max = 24,6; mean = 22,9; SD = 1,53). G3 consisted of five women aged 18 to 36 years (mean =27. 8; SD = 6.65). The controlled independent variable was related to skin phototype (dark brown to black skin).

The age range of the three groups was defined between 18 and 50 years of age, used to exclude, at the lower limit, interference from vocal changes, and presbyphonia at the upper limit. The groups were matched according to age (p=0.057)^(19,20)^.

The exclusion criteria were pregnancy, glaucoma, undiagnosed lesion on the area to be irradiated or close to it, changes in the thyroid gland, infection at the site of application, history of cancer or photosensitivity^(21)^. Due to structural and size differences in anatomical dimensions between men and women, the present study was carried out only with female participants.

The participants who had a positive self-perception of their vocal quality (reported having a good or very good voice), and self-reported absence of vocal symptoms (fatigue and/or discomfort), were invited to undergo a speech-language and otorhinolaryngological evaluation. Speech-language assessment consisted of auditory-perceptual analysis of the general degree of dysphonia (G) on a four-point scale, in the tasks of habitually sustained vowel /a/ and connected speech (days of the week). The speech-language evaluation was carried out by one of the researchers, with experience in auditory-perceptual analysis. Participants with neutral vocal quality (G0) were included.

The otorhinolaryngological evaluation was performed by an otorhinolaryngologist, using a flexible nasofibrolaryngoscope. We considered with a normal laryngeal exam those who presented no lesions in their VF and had complete glottal closure. A posterior triangular cleft in women was considered normal.

To determine the BMI, we calculated body mass (BM), divided by height (St) squared (BM/Hgt.^2^). The BMI of reference are based on the World Health Organization (WHO) classification (underweight <18.5; normal range: 18.5-24.9; overweight: ≥ 25; pre obese 25.0-29.9; obese class I 30.0-34.9; obese class II 35.0-39.9; obese class III ≥40). Based on this calculation, it was possible to classify the nutritional status of women, using cutoff points established in the literature (BMI ˂ 25: normal weight; BMI ≥ 25: overweight)^(15,16)^. The participants underweight and obese class III were not included in this study.

To determine the skin phototype, all participants were classified according to the Fitzpatrick Scale through self-assessment of solar sensitivity based on six phototypes of skin tones ranging from very light (skin type I) to very dark (skin type VI)^(18)^.

### Identification of anatomical points on the neck

At first, approximate anatomical limits were established by locating the glottic level and the main intrinsic muscles of the larynx, according to anatomical studies and references from otorhinolaryngological procedures such as thyroplasty and laryngeal electroneuromyography^(22)^. The main structures of the neck were palpated to identify: 1) the midline thyroid cartilage; 2) the hyoid bone; 3) the thyroid cartilage, its notch and its lower edge; 4) the cricothyroid space and; 5) upper border of the cricoid cartilage.

To identify the muscles, we drew an identification line between the thyroid notch and the lower border of the thyroid cartilage. This distance was measured by establishing the midpoint, and from it, six points were identified.

**Point 1** was defined as the midpoint between the thyroid notch and the lower border of the thyroid cartilage, aiming at the topography of the anterior VF commissure.

From the midpoint of the anterior height of the thyroid cartilage, another point was traced 1 centimeter (cm) perpendicular and lateral to the midline, called Point 2, and a third point, 2 cm from the midline, was defined and named Point 3. **Point 2** was at the level of the membranous part of the vocal fold in the region of the thyroarytenoid muscle (TA) and the epithelium layer of the vocal fold; and **Point 3** in the cartilaginous part of the vocal fold in the region of the lateral cricoarytenoid muscle (LCA).

**Point 4** was defined as the point with lateral extension at the middle height of the thyroid cartilage and anterior margin of the sternocleidomastoid muscle, aiming to reach the posterior third of the lateral lamina of the thyroid cartilage. This topography was established due to reports of frequent use of laryngeal photobiomodulation in speech therapy clinical practice.

**Point 5** was defined as the point in the cricothyroid space at 1.5 cm from the midline, aiming to be in the topography of the cricothyroid muscle (CT).

**Figure 1:**
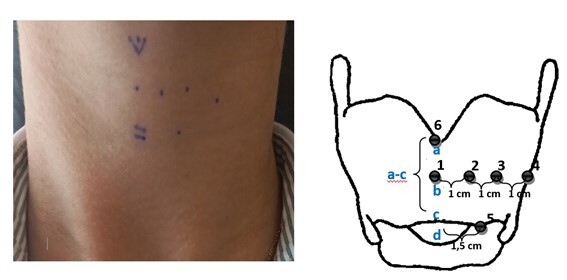
Schematic representation of the cervical markings for the Laser light application

Finally, **Point 6** was defined just above the thyroid notch, to assess the anatomical structures that can be reached from this stimulation point, since it is routinely used in clinical practice by many speech therapists. Markings were performed and tested in only one hemilarynx, due to exam discomfort. Figure 1 depicts the schematic representation of the markings that were considered to establish the anatomical location of the intrinsic muscles of the larynx.

### Visual monitoring by nasofibrolaryngoscopy

After establishing the laryngeal anatomical points and its landmarks, the LASER light was employed to the six points established on the neck, on the left side of the larynx. The LLLT was used concomitantly with nasofibrolaryngoscopy, to monitor the reach of light into the larynx and check whether the location of the established points was reliable for reaching the aimed laryngeal intrinsic muscles.

The laryngeal exam was performed by an otorhinolaryngologist using a Machida® flexible nasopharyngolaryngeal endoscope with a 3.2 mm diameter and a Ferrari® halogen continuous light source. After seating the individual with the head erect and comfortable, the optical fiber was introduced through the nasal cavity after nasal vasoconstriction with oxymetazoline. Progression of the flexible optics was performed until obtaining a satisfactory image of the larynx. The images were recorded and stored in digital format and made available to the participants. For the LASER application; DMC equipment, model Therapy EC, with 100 mW of power and output spot with an area of 0.028 cm^2^ in the red wavelength at doses of 3J, 6J and 9J. Its application was punctual and with skin contact, along the assessed muscles, with three applications per point, considering the different doses.

The LASER was applied by a speech therapist concomitantly with the laryngeal examination. Following the manufacturer's recommendations and meeting the safety standards established by the National Health Surveillance Agency for low-level LASER equipment, the researchers responsible for the application and the participants wore protective glasses throughout the procedure.

### Data analysis

In order to analyze the reach of the LASER light into the anatomical structures of the larynx, based on nasofibrolaryngoscopy monitoring, two researchers of the study (speech therapist and otorhinolaryngologist) ran a quantitative and qualitative analysis of the data. During all applications, at different dosages (3J, 6J and 9J), the nasofibrolaryngoscopy exams were recorded and the researchers visually analyzed the images, reaching a consensus.

The first analysis considered the reach of the LASER light (present/absent) into the larynx, at the time of application, at different doses. Upon visualizing the LASER light in the larynx, it was considered present, and when it was not visualized, absent, for each of the doses. At this moment of the evaluation, the examiners also identified the anatomical structure of the larynx on which the light shone.

The second evaluation analyzed the degree of LASER light shone, when present in the larynx, and considered, for each dose, the following results: 1) Very strong (visually intense); 2) Strong (visually strong); 3) Weak (visually present, but weak); 4) Very weak (visually very weak, almost not noticeable). The graduation related to the perception of light in the larynx was carried out in a qualitative and comparative way among the participants.

We ran the statistical analysis of the data using the MINITAB statistical program, version 17. First, we made a descriptive analysis of the data with measures of central tendency and dispersion, as well as the calculation of absolute and relative frequencies. Subsequently, we used the Anderson-Darling test to verify sample normality for numerical variables. To compare the age, height, BMI and weight variables between groups, the ANOVA parametric test with repeated measures was used. For the analysis of categorical variables (presence/absence and degree of illumination of the LASER light), the chi-square test was used. For the analysis of the degree of light illumination, the results were categorized into strong (very strong and strong) and weak (weak and very weak), with the exception of points 3, 4 and 6 where the analysis was not possible due to the sample size. In all analyses, a significance level of 5% was considered.

## RESULTS

The average height of the 15 participants was 1.60 meters (m) (minimum: 1.45; maximum: 1.72; SD=0.67) and weight 66.4 kilos (Kg) (minimum 48; maximum 94; SD=11.3). The average BMI value was 25.8 (minimum: 19.7; maximum: 36.7; SD=3.85). G2 presented higher values of weight (p-value = 0.000) and BMI (p-value=0.000), when compared to G1 and G3 (Table 1).

**Table 1:** Height, BMI, and weight measurements in Groups I, II and III

	Groups	n	Min	Max	Mean	SD	P value
Height (m)	Group 1 (light skin /BMI<25)	5	1.60	1.70	1.64	0.04	0.466
	Group 2 (light skin /BMI>25)	5	1.55	1.59	1.63	0.11	
	Group 3 (dark skin /BMI<25)	5	1.55	1.63	1.59	0.08	
BMI (Kg/m^2^)	Group 1 (light skin /BMI<25)	5	19.7	25	22.4	1.86	0.000
	Group 2 (light skin /BMI>25)	5	29.5	36.7	32.0	9.20	
	Group 3 (dark skin /BMI<25)	5	20.5	24.6	22.9	1.53	
Weight (Kg)	Group 1 (light skin /BMI<25)	5	55	67	59.8	5.36	0.000
	Group 2 (light skin /BMI>25)	5	71.7	94.0	81.4	7.92	
	Group 3 (dark skin /BMI<25)	5	48.0	63.0	58.2	5.17	

**Caption:** SD = standard deviation; Kg = kilograms; m = meters; Max = maximum; Min = minimum; n = number; Group 1 = light skin/BMI<25; Group 2 = light skin/BMI >25; Group 3 = dark skin/BMI <25

According to the Fitzpatrick scale, in groups G1 and G2, 40% of the participants had Type I skin color (white skin), 40% type III (light brown skin) and 20% type II (white skin). In the G3 group, 40% had phototype IV (moderate brown skin), 40% type V (dark brown skin) and 20% type VI (dark skin).

The mean distance between the thyroid cartilage notch and its lower edge in the sample was 21 millimeters (mm) (minimum: 18 mm; maximum: 28 mm; SD=3.4 mm) and the midpoint was 10 mm (minimum: 9 mm; maximum: 14mm; SD=1.8). The values referring to the laryngeal distance measurements for each group are described in Table 2.

**Table 2:** Laryngeal measurements in the groups I, II and III

Distance (mm)	G1				G2				G3			
	Min	Max	Mean	SD	Min	Max	Mean	SD	Min	Max	Mean	SD
a-c	18	25	21	0.56	20	27	23	0.51	18	28	21	0.4
b	9	13	10	0.17	10	13	11	0.1	9	14	10	0.2

Caption: a-c = distance between the thyroid cartilage notch and its lower edge; b = midpoint; cm (centimeters); SD = standard deviation; G1 = light skin/BMI<25; G2 = group 2- light skin/BMI>25; G3 = dark skin/BMI<25; Max = maximum; Min = minimum

Table 3 presents the analyzes of the presence/absence of LASER light in the larynx, with the frequency of occurrence and the anatomical structure affected by the light.

**Table 3:** Occurrence of incidence of light on anatomical points

Anatomical points	Group 1		Group 2		Group 3		p-value
	present (%)	absent (%)	present (%)	absent (%)	present (%)	absent (%)	
Point 1	5 (100%)	0 (0%)	5 (100%)	0 (0%)	5 (100%)	0 (0%)	---
Point 2	5 (100%)	0 (0%)	5 (100%)	0 (0%)	5 (100%)	0 (0%)	---
Point 3	3 (60%)	2 (40%)	1 (20%)	4 (80%)	3 (60%)	2 (40%)	0.322
Point 4	2 (40%)	3 (60%)	1 (20%)	4 (80%)	0 (0%)	5 (100%)	0.194
Point 5	5 (100%)	0 (0%)	5 (100%)	0 (0%)	5 (100%)	0 (0%)	---
Point 6	0 (0%)	5 (100%)	2 (40%)	3 (60%)	3 (60%)	2 (40%)	0.060

Caption: Point 1 = anterior commissure of the vocal folds; Point 2 = membranous part of the vocal folds (TA); Point 3 = Cartilaginous portion of the vocal folds and region of the lateral cricoarytenoid muscle (CAL); Point 4 = posterior third of the thyroid lamina; Point 5 = Cricothyroid muscle (CT); Point 6 = thyroid notch. Group 1 (light skin/BMI<25); Group 2 (light skin/BMI >25); Group 3 (dark skin/BMI <25)

The results suggest that the light focused on the region of the anterior commissure of the VF (Point 1), and on the TA (Point 2) and CT (Point 5) muscles, in 100% of the participants, in the three studied groups. Regarding the dose, light was considered present in the three doses analyzed (3J, 6J and 9J).

In the evaluation of Point 3, the anatomical region that the LASER light fell on was the cartilaginous part of the vocal fold (LCA muscle), in seven (46.7%) participants, and in relation to the group, the results of presence/absence of light in the larynx, had similar behavior, suggesting that the BMI (G2) and phototype (G3) variables did not interfere in the results (p-value=0.322). The dose also did not interfere with the presence/absence of light in the larynx.

Regarding Point 4, the light was visualized in the region of the piriform sinuses in 20% of the sample (three participants), and the results did not differ in relation to the groups (p-value=0.194) and the evaluated dose.

Point 6 was visualized in the supraglottic region (epiglottis) of five participants (33.4%). Neither the groups interfered in the results (p-value=0.060) nor the evaluated doses.

**Figure 2:**
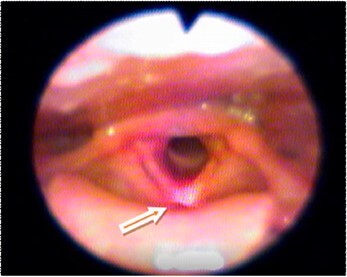
Incidence of the LASER light on Point 1 (anterior commissure) – light irradiates on the anterior commissure of the vocal folds

**Figure 3:**
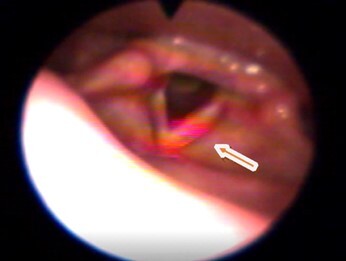
Incidence of the LASER light on Point 2

**Figure 4:**
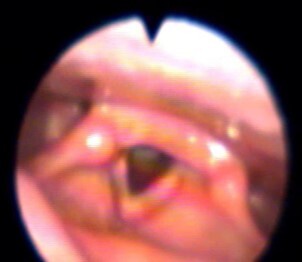
Incidence of the LASER light on Point 3

**Figure 5:**
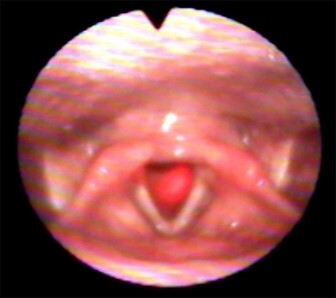
Incidence of the LASER light on Point 5

Figures 2 to 5 illustrate the reach of the LASER light in the different anatomical points evaluated. The light shone on the anterior commissure of the VF, when applied to Point 1 (Figure 2), in the membranous portion of the vocal fold (TA muscle and the superficial mucosa of the VF), when applied to Point 2 (Figure 3) and, in the cartilaginous portion of the vocal fold in point 3 (Figure 4).

The light was visualized more in the middle and posterior region of the glottis, when applied to Point 3 (Figure 4) and in the piriform sinuses when the application occurs at Point 4.

When applying the low level laser therapy at Point 5 (Figure 5), the light radiates to the subglottic region in the paramedian line. When applied to Point 6, the light is visualized in the supraglottic region, at the level of the epiglottis, in some individuals. Points 4 and 6, when present, showed weak light intensity in the larynx, making their illustrations difficult.

Table 4 presents an analysis of the LASER light illumination degree in the larynx, when it was present, for the three evaluated groups.

**Table 4:** Degree of illumination (brightness intensity) at each anatomical point by group

	Degree of illumination	Group 1	Group 2	Group 3	p-value
Point 1	Very strong/Strong	5 (100%)	0 (0%)	5 (100%)	0.000
	Weak/Very weak	0 (0%)	5 (100%)	0 (0%)	
Point 2	Very strong/ Strong	1 (20%)	0 (0%)	4 (80%)	0.011
	Weak/Very weak	4 (80%)	5 (100%)	1 (20%)	
Point 3	Very strong/Strong	0 (0%)	0 (0%)	0 (0%)	---
	Weak/Very weak	3 (100%)	1 (100%)	3 (100%)	
Point 4	Very strong/Strong	0 (0%)	0 (0%)	0 (0%)	---
	Weak/Very weak	2 (100%)	1 (100%)	0 (0%)	
Point 5	Very strong/Strong	5 (100%)	3 (60%)	5 (100%)	0.70
	Weak/Very weak	0 (0%)	2 (40%)	0 (0%)	
Point 6	Very strong/Strong	0 (0%)	0 (0%)	0 (0%)	---
	Weak/Very weak	0 (0%)	2 (100%)	3 (100%)	

Caption: Point 1 = anterior commissure of the vocal folds; Point 2 = membranous part of the vocal folds (TA); Point 3 = Cartilaginous portion of the vocal folds and region of the lateral cricoarytenoid muscle (CAL); Point 4 = posterior third of the thyroid lamina; Point 5 = Cricothyroid muscle (CT); Point 6 = thyroid notch. Group 1 (light skin/BMI<25); Group 2 (light skin/BMI >25); Group 3 (dark skin/BMI <25)

At Point 1, light was present in the three evaluated groups (n=15). In G1 (light skin and BMI below 25) and in G3 (dark skin and BMI below 25) the light showed strong illumination in all participants, being strong in 100% of the group in G1 and very strong (80%) and strong (20%) in G3, respectively. The results suggest that BMI and phototype interfere with the degree of LASER light illumination in the larynx (p-value=0.000). The increase in BMI decreases the degree of light illumination in the larynx, while the moderate to black skin phototypes increase the light illumination.

Point 2 (TA) was also visualized in the entire sample (n=15), with different degrees of illumination per analyzed group. In G1, the degree of illumination in most of the participants was weak (80% weak and 20% strong), in G2 it was very weak (100%). In G3 it was strong (80%). The results indicate that the BMI decreases the degree of illumination and phototypes IV to VI increase (p-value=0.011).

In the evaluation of Point 3, light was identified as present in seven participants, distributed as follows: G1 (n=3); G2 (n=1) and; G3 (n=3). Of these, the degree of illumination ranged from weak (G1 and G3) to very weak (G3).

At Point 4, lighting was present in three participants, all with very low lighting: G1 (n=2) and G2 (n=1).

At Point 5, light was present throughout the sample (n=15). The degree of illumination was strong in all participants in G1 and G3 (100%), and in most of G2 individuals (60%). BMI and phototype did not seem to interfere with the degree of illumination at Point 5 (p-value = 0.070).

Light was present in only five participants in the evaluation of Point 6, two in G2 and three in G3. In these, the degree of illumination was weak (G2 and G3).

Regarding the analysis of the doses (3J, 6J and 9J), the degree of light illumination seems to increase as the dose applied to the larynx increases, in the different anatomical points evaluated.

Knowledge of the laryngeal anatomy, with attention to its framework and the functional dynamics of its extrinsic and intrinsic musculature is extremely important in laryngological therapies, not only with the aim of avoiding complications, but also to envision its therapeutic potential when necessary. In clinical practice, establishing the neck anatomical points that correspond to the laryngeal musculature and its glottic level are important in neck surgeries, such as in thyroplasties, in laryngeal injections with external neck access, in laryngeal propedeutics, such as in electroneuromyography and image exams, and also for speech therapy^(22-30)^. Thus, precision in relation to superficial location in correspondence with the intrinsic muscles of the larynx is essential.

Since LLLT is used to enhance muscular action, this study aimed at the intrinsic muscles of the larynx. However, as it is possible to visualize red light of the LASER in tissues using videolaryngoscopy, it can be inferred that its action also affects the mucous layer of the vocal fold, with possible repercussions on its phonatory dynamics^(8,9)^.

In Brazil, the increasing use of LASER in vocal clinics increases the need for standardization of application sites since the points used are empirical and vary between speech therapists^(31)^. At the moment, there are expert opinions that recommend the following application sites: 1) point just above the thyroid notch; 2) lateral points in the most superior region of the thyroid cartilage; 3) midpoint between the thyroid notch and the lower edge of the thyroid cartilage; 4) point 10 mm lateral to the midpoint of the thyroid cartilage height; 6) point on the lower border of the thyroid cartilage in the midline.

As a reference for measurements in our population, we used the study by Jotz et al.^(22)^. The authors analyzed 50 Brazilian female larynges with metric evaluations. The results of measurements in millimeters in women were: Thyroid angle in women: 91.08+/- 13.44; Thyroid cartilage thickness: 27.51 +/- 3.44; distance between thyroid notch and anterior commissure: 7.88 +/- 2.28; distance between anterior commissure and lower border of the thyroid cartilage 7.49 +/- 2.57; total extension of the vocal fold: 17.91 +/- 2.15; membranous part of the vocal fold 11.17 +/- 1.68; cartilaginous part of the vocal fold: 6.73 +/- 1.13; vocal fold lateral thickness: 5.03 +/- 1.10; vocal fold depth: 8.61 +/- 1.48. The authors observed that the anterior commissure in women was found slightly below the midpoint of the thyroid height^(22)^.

Based on the searched literature, the anatomical markings of six points in the neck region were defined for access to the intrinsic muscles of the larynx and the vocal folds themselves^(22-25,32-34)^. We will describe below how these topographies were established.

### Target topography: glottic level and anterior commissure

The location of the glottic level has long been studied because, as it is protected by the thyroid framework, its external access is easily obtained by opening a window in the cartilage. Such access is widely used in type 1 thyroplasty with the objective of medialization of the vocal folds, initially described by Isshiki et al.^(25)^. Such surgical reference corresponds to the anatomical reference of the glottic level and consequently of the thyroarytenoid musculature and anterior commissure, and from this reference it was possible to define points 1 to 4^(25-27,32-34)^.

Thus, **Point 1** was standardized at the midpoint between the thyroid notch and the lower border of this cartilage. The average distance between the thyroid cartilage notch and its lower border found in the measurements of this study was 21 mm and the literature describes an average value of 15.39 +/- 2.3 mm in the female Brazilian population^(22)^. Other descriptions in the literature are measurements of 18.25 mm (Ortug, 2021)^(24)^, 13.23 mm (SD 1.37) (Cinar 2003)^(32)^ and 14.4 m (SD 2.92) (Enver 2018)^(33)^, all studies carried out in Turkey. German studies carried out with European Causasians found values of 15.8 mm (SD 1.07) (Eckel 1994)^(23)^ and 15 mm (SD 2.1) (Sprinzl, 1999)^(34)^ as the distance from the thyroid notch to the lower border of the thyroid. All studies to establish the metric parameters of the larynx were performed on cadavers using a caliper (precision ruler) with direct measurements on the dissected parts. The results of this study regarding the distance between the thyroid notch and the lower border of this cartilage were greater than the references in the literature. This difference can be explained by the fact that this is a study in patients with neck marking through digital palpation with loss of exact definition of the cartilaginous borders. Therefore, it is important to consider that the superficial skin layer is flexible and that superficial neck markings are approximate. As this distance glimpsed the marking of the midpoint of the thyroid length, this difference between the values did not interfere with the visual assessment of the larynx by endoscopy, since point 1 was consistent with the level of the anterior commissure, and the midpoint of the thyroid cartilage height found in this study was 10 mm.

In type 1 thyroplasties, external access is performed to the thyroid cartilage, opening it with removal of a cartilage window at the level of the vocal fold. According to Isshiki^(27)^, the anterior commissure projects externally to the thyroid, at the midpoint between the distance of the thyroid notch and the lower border of the cartilage, with a maximum error of 2 to 3 mm in men and 1 to 2 mm of error in women. Other studies found that the anterior commissure in women is located a little below the midpoint^(22,24)^ or, on the contrary, 0.5 to 3 mm above this distance^(32)^. Therefore, despite the midpoint of the thyroid cartilage being the reference to the location of the anterior commissure, this topography may vary slightly along the laryngeal vertical axis.

### Targeted topography: membranous and cartilaginous portions of the vocal fold and region of the lateral cricoarytenoid muscle (LCA)

In surgeries for medialization of the vocal folds, called type I thyroplasties, to locate the vocal fold and its medialization in its membranous extension, a horizontal incision is made starting from the midline of the thyroid cartilage, making a rectangle with its anterior edge 5 mm laterally from the midline and lower border 5mm away from the lower border of the thyroid^(25-27)^. Knowing that the vocal fold extends laterally to the anterior commissure, following the thyroid cartilage, we defined points 2 and 3. Due to the size of the TA muscle, we defined **Point 2** at one centimeter perpendicular and lateral to the midline and **Point 3** at two centimeters from the midline, to cover the entire glottic extension. As the reference for the vocal fold length in the larynges of Brazilian women is 17.91mm (SD 2.15) in total length, mean length of the membranous part of 11.17 mm (SD 1.68) and cartilaginous part 6.73 mm (SD 1.13)^(22)^, placing the red light 10 mm laterally at the level of the anterior commissure would be an adequate distance for its location in the laryngeal lumen at the glottic level in the membranous part, but 2 cm laterally would exceed the vocal extension based on Jotz measurements^(22)^. The results are compatible with these metrics, since in only seven participants the red light was visualized at the glottic level. Due to the coverage area of the LASER light, it can be inferred that some part of the LCA could be being reached, as this muscle is located slightly lower, more lateral and more posterior to the TA. With the definition of points 1, 2 and 3, it was intended, with the application of the red light of the LASER, to reach the entire extension of the VFs, from anterior to posterior.

### Targeted topography: cricothyroid muscle

Regarding **Point 5**, whose target was the CT muscle, the reference in the literature for its location via electroneuromyography is the cricothyroid space^(28-30)^. In this test, a needle is introduced 0.5cm from the line at an average angulation of 30 to 45 degrees laterally, with the introduction of 15 to 20mm of the needle electrode. The CT is located 1cm deep under the skin^(29,30)^. Thus, we defined Point 5, located 1.5 cm from the midline in the cricothyroid space, aiming to reach the CT.

### Evaluation of the areas reached by the red laser light in the posterior third of the thyroid lamina and in the anterior border of the sternocleidomastoid muscle

**Points 4 and 6** were tested based on the recurrent recommendation of specialists on the use of photobiomodulation in speech therapy clinical practice. These two anatomical landmarks were defined based on empirical data, and do not have a specific target musculature based on findings in the literature. The purpose of the study was to verify which structures would be illuminated with the projection of red LASER light at these points on the neck.

### Correspondence of the red LASER light with the laryngeal anatomy and variables that impact its visualization

From the visual monitoring of the LASER light scope established by nasofibrolaryngoscopy, it was possible to observe that, at Point 1, the red light focused exactly on the anterior commissure in all videolaryngoscopies performed (100%). There was also total correspondence of Point 2 with the reach of the red light, and in all cases, it was visible at the glottic level, corresponding to the TA muscle. These results suggest that these topographies are the most consistent for the application of LASER. At these points, group II (high BMI) presented a very weak degree of illumination, while group III a very strong degree of illumination (phototypes IV to VI), suggesting that both the increase in body mass and the skin phototype impact visualization of the LASER red light in the larynx when seen by videolaryngoscopy.

In Point 3, the red light was visualized in the posterior third of the VFs (cartilaginous part of the vocal fold) in seven participants (46.7%) of the three studied groups, and in all of them, the degree of light illumination was classified as weak or very weak. Such results suggest that due to the total length of the vocal fold being approximately 17.91 mm (+/- 2.15),^35^ the 2 cm lateralization may have exceeded the total length of the vocal fold, or there may be variation in neck thickness in the anteroposterior direction, decreasing the access frequency of the LASER light in the membranous part of the vocal folds, starting at Point 3, when red light is used.

Upon analyzing the LASER light reach in the anatomical marking of Point 4, only in three participants (20%) a weak light was visualized in the piriform sinus region, its action on the intrinsic muscles of the larynx being questionable.

In evaluating the reach of the light in the topography of Point 5, the red light was very clearly reaching the subglottic region at the level of the cricothyroid muscle in all participants of the study (100%), with the degree of light illumination ranging from very strong to strong in the majority of the sample in the three groups. Such results suggest that the cricothyroid space point enables reaching the target muscle (cricothyroid muscle), and that BMI and skin phototype do not seem to influence the degree of LASER light illumination. Probably, because point 5 is positioned in the cricothyroid space and does not have a cartilaginous framework interposing the skin and the laryngeal lumen, the light intensity was very evident in this topography, without significant influence of the other physical barriers analyzed.

Penetration of the red LASER light was visualized in the videolaryngoscopy examination in five (33.4%) of the participants when the light was applied at point 6. As such topography is just above the thyroid cartilage and corresponds to a level superior to the level of the vocal folds, the red light focused on the supraglottic region, therefore, such topography is not recommended for the use of LASER in the larynx.

One of the discussions regarding photobiomodulation is related to the penetration of visible and non-visible light spectra in tissues, in relation to their range in depth and dispersion. In the application of LASER on the skin surface, approximately 4-7% of the incident light is lost in the stratum corneum due to the refraction of light beams, the remainder penetrates the skin and subcutaneous tissue reaching deeper layers^(5,35)^.

In cases where the target tissue is not cutaneous, for a positive clinical effect to occur, the delivery of energy to deeper tissues must overcome the barriers of the skin, which has photoprotective and dispersing properties. Regardless of the target tissue, knowing how much the skin can interfere with the process of light distribution in tissues has important clinical implications, including the selection of irradiation parameters for each patient, considering tissue characteristics (depth and conditions of the treated tissue), sex, age, anatomical location of the tissue and phenotypic characteristics^(36)^. The optical properties of the skin are affected by the concentration of chromophores that are highly dependent on wavelength and, when passing through the skin, the light undergoes multiple scattering and absorption along the way to the treated tissue^14)^.

Individual characteristics must be considered for adjusting the dosimetric parameters for the LASER application, with melanin and subcutaneous fat being one of the main structures that interfere with the absorption and scattering coefficients of the skin and, consequently, with the light absorption in superficial tissues^(15,17,35,36)^. Individuals with darker skin have a higher density of melanin particles, their particles being smaller than Caucasians, being more effective in light dispersion, thus reducing the penetration of light into deeper tissues. Souza-Barros et al.^(35)^ when analyzing the effects of skin color and skin thickness on transmittance and reflectance with the use of LASER, found that transmittance was significantly lower in darker skins when considering red and infrared light, when evaluating a same skin thickness. When skin thickness increased, effects related to skin color became less important. In this study, the authors discuss the individualization of the diode LASER doses adjustments to compensate for the photocutaneous effects, increasing the dose in dark skin by 33% when using infrared light and by 45% when using red light^(35)^. Nussbaum and Zuylen^(37)^ when analyzing the transmittance of light in skin folds, found that the transmittance of red light (660nm) decreased more significantly than that of infrared light (840nm) when analyzed in darker skins. On the other hand, transmittance values decreased exponentially when skin thickness increased in both light spectra (red and infrared light).

## FINAL REMARKS

The findings from this study reinforce the hypothesis that neck structures impact the penetration of LASER light. Physical barriers such as subcutaneous tissue thickness, related to the amount of body fat and directly proportional to BMI, impacted the intensity of light perceived in the pharyngolaryngeal cavity, decreasing the intensity of red light. The WHO endorsed the BMI as a anthopometric measurement correlated to cardiovascular risk^(16)^. In order to reduce data variability, there were no underweight (BMI < 18.5) or obese class III (BMI ≥ 40) participants. However, future studies are needed to verify the light penetration in those groups, that are at opposite ends of the subcutaneous tissue thickness.

The Fritzpatrick scale is based on a person’s tendency to sunburn and ability to tan. It was adopted as an indirect reference in the melanin concentration in the skin of participants. Although the Brazilian population is heterogenic in its ethnical composition, this scale can be applied in this sample as it is not related to race.

Melanin is the main pigment on the surface of vertebrates, protecting the skin from the effects of sunlight^(17)^. In this present study, individuals with darker skin had a more evident intensity of red light. Contrary to expectations, the light was more intense and more dispersed in the videolaryngoscopy images. Some of the hypotheses raised are that melanin could alter the refraction of LASER rays by increasing the visualized area of the emitted light, another hypothesis is that there are other elements that interfere in this interaction. In addition to melanin, skin color is determined by hemoglobin, bilirubin and carotenoids^(17)^. Furthermore, the anatomical structures of the neck framework can influence the LASER light penetration, such as the thickness of the extrinsic muscles and the thyroid cartilage, which is a cartilage that protects the larynx as a whole. Studies could also assess how much this cartilage interferes with the penetration of LLLT, either due to its thickness or its degree of calcification, which increases according to the individual's age.

Connective tissue and muscles also absorb light and function more as a “photon sink” which could also be a variable for the change in the LASER transmittance pattern as deeper tissues with several layers between the skin surface and the laryngeal lumen^(35)^. These other variables were not measured in this study and could also interact with red light, altering its penetration into tissues. Further studies are needed to assess the real impact of each variable on the effects of LASER and whether individualization of the applied dose would be necessary to compensate for these barrier effects.

Regarding the application of different doses (3J, 6J and 9J), these seem to influence more on the degree of light illumination, with a directly proportional relationship, that is, increasing the dose increases the degree of illumination.

The magnitude of the biostimulatory or bioinhibitory effect attributed to low level laser therapy when interacting with biological tissues is determined by parameters such as dose, wavelength and irradiation points, considering the existence of a dose-dependent relationship to trigger a biological response^(2)^.

In this present study, the LASER was applied at one point per target muscle with a total dose per hemilarynx between 18J and 54J at the six points evaluated. The literature shows that 75% of the doses that showed positive results for improving performance in large muscle groups (e.g. quadriceps) were between 60J and 300J and that 85% of the doses that showed positive results in small muscle groups (e.g. biceps) were between 20J and 60J^(38)^.

The number of points used in the literature varied between two and 29, depending on the extent of the muscle group studied, and the most used doses per point were 7J and 30J^(39)^. Considering the extent of the intrinsic muscles of the larynx, we consider that only one point per target muscle is the most indicated. We still do not know the optimal dose in the larynx capable of generating interaction between the target muscles and the applied light, considering the depth and extension of the muscles.

Regarding the area reached by photobiomodulation, according to the LASER equipment Manual, DMC, model Therapy EC, the area of action of the light rays would be 1 cm in diameter, which goes beyond the contact area of the device with the skin. Thus, although we aimed at the intrinsic laryngeal musculature, small deviations for the location of the target musculature (provided that within the range of action of the device) would be tolerable and would allow small deviations and anatomical variations.

We suggest that the professional speech therapist, when establishing the application points marked with a pen on the skin, preserve the position of the patient's head preferably in a neutral position, as changing the position of the head could shift the markings outside the anatomical reference points. In this way, we suggest the neck landmarks must be palpated again, before the application, to guarantee that the LASER is applied in the standardized points.

Although the target is the intrinsic muscles of the larynx, the external application of LASER reaches all layers that are exposed to photobiomodulation, such as the skin, subcutaneous tissue and neck muscles. The extrinsic musculature of the larynx is important to keep the laryngeal skeleton stable and to produce a symmetrical vibration of the VFs. Thus, the cutaneous application of the LASER will also reach the extrinsic musculature, generating a probable increase in its metabolism and muscle relaxation, which may be favorable for improving the vocal quality of individuals with dysphonia and muscular hyperfunction in the neck.

Other studies are necessary to analyze function improvement in these extrinsic muscle groups with the use of LASER and to evaluate which would be the most indicated dosages.

We emphasize that this study presents preliminary results to establish the anatomical location of the intrinsic muscles of the larynx for the application of low level LASER, but we still do not know which doses present better interaction with the target muscles in different situations in the voice clinic.

The basic science of photobimodulation involves knowledge of chemistry, physics, and biology, and therefore, we cannot simplify biological processes. It is difficult to speculate the reach of light in a specific location, in addition to the depth of the skin.

This study is the first proposing to evaluate the reach of red light in laryngeal structures, as well as to evaluate light intensity and variables associated with tissue penetration capacity. This research provides valuable starting parameters for investigation and better elucidation of the interference of skin phototype and fat percentage variables in the selection of the best irradiation parameters.

Based on scientific literature, this study focused on two variables that could interfere with the use of photobiomodulation: the thickness of the subcutaneous layer reflected by the BMI and the concentration of melanin related to the skin phototype. The option of not having a group with dark skin and BMI>25 would be to avoid creating a confounding variable, by having two variables together that could interfere with the visualization of the LASER. Therefore, the difference between group 1 (light skin and BMI<25) and the other groups would be due to only one variable, BMI or dark skin. Future studies are needed to elucidate the interaction of the photobiomodulation in the individuals with dark skin and overweight.

Finally, as this is a study with a limited sample size, further studies with larger and different samples, such as men or groups with variations in subcutaneous thickness, with different BMI patterns and with different skin tones, would need to be studied. Therefore, future studies are needed to determine the ideal range of LASER doses and the main anatomical points of application in males.

## CONCLUSION

The present study suggests that the LASER red light focuses on the anterior commissure of the vocal folds (Point 1), and on the topography corresponding to the TA muscles (Point 2) in the CT (Point 5) at doses of 3J, 6J and 9J, with interference from BMI and skin phototype.

In the topography corresponding to the cartilaginous part of the vocal folds and the LCA (Point 3) the LASER will benefit around half of the participants when using red light, and its use is debatable within clinical practice.

The evidence that the low light LASER reaches the larynx in specific anatomical points may provide direction for the standardization of its use in voice practice. Therefore, anatomical points with no correlation with the larynx and which are widely used in clinical practice may also have their use discontinued.

At the dose used in this study, BMI and skin phototype suggest interfering with the degree of illumination of the red light of the LASER when it is applied to the larynx. The degree of light illumination increases with the increase in the dose applied to the larynx (3J, 6J and 9J). The increase in BMI decreases the degree of light illumination in the larynx, while the moderate to black skin phototype increases the light illumination.

In clinical practice, these variables need to be considered when prescribing the irradiation parameters used in photobiomodulation, as well as whether the application position in the neck should be taken into account so that the laryngeal muscles are better stimulated and a better effect of the LASER is obtained in the larynx.
